# The intricate interplay between circadian rhythm, androgen signaling, hormone therapy, and cellular senescence in prostate cancer

**DOI:** 10.1007/s10555-025-10302-1

**Published:** 2025-11-20

**Authors:** Mehdi Heidari Horestani, Aria Baniahmad

**Affiliations:** Present Address: Institute of Human Genetics, Jena University Hospital, Friedrich Schiller University, Am Klinikum 1, 07740 Jena, Germany

**Keywords:** Cancer, Prostate cancer, Androgen receptor, Circadian rhythm, Clock genes, Cellular senescence

## Abstract

Prostate cancer (PCa) is the second most diagnosed cancer and the fifth leading cause of cancer death among men worldwide. Androgen receptor (AR), as a ligand-activated transcription factor, is important for both prostate development and PCa progression. Understanding the molecular mechanisms of prostate carcinogenesis has led to the development of therapeutic strategies targeting AR. Inhibiting AR is currently the gold standard for hormone therapy. However, eventually resistance to therapy occurs. The activation of AR by supraphysiological androgen levels (SAL) used currently in clinical trials paradoxically also inhibits PCa progression and induces cellular senescence. Interestingly, circadian rhythm controls hormone biosynthesis including androgens. Intriguingly, SNPs in several clock genes have been associated with PCa risk linking increased cancer risk with day-night shifts. Here, we discuss whether the efficacy of hormone therapeutics depends on the biological clock. It emerges that androgens control the expression of clock genes also intersecting with SAL-induced cellular senescence suggesting a complex and understudied network that governs PCa progression. This review highlights the multifaceted roles of AR signaling in PCa, emphasizing its ability to promote cellular senescence by AR-targeted therapy via genomic and non-genomic pathways and crosstalk with the regulation of circadian clock genes. The intricate interplay between circadian rhythm, androgen signaling, and cellular senescence presents a promising yet underexplored research area in PCa and suggests a multilayered regulatory network that could shape PCa progression and treatment outcomes. Unraveling this network may uncover novel chronotherapeutic strategies and provide new insights into disease, prognosis, and therapy options.

## Introduction

The prostate is a male reproductive gland about the size of a walnut, situated underneath the bladder. It produces prostatic fluid, which plays a crucial role in nourishing and transporting sperm [1]. Prostate cancer (PCa) is the second most commonly diagnosed cancer and the fifth leading cause of cancer-related deaths among men globally, with an estimated 1.46 million new cases and 396,000 deaths in 2022 [2]. Although mortality rates have declined in some countries, the global burden of PCa is expected to rise due to population aging and the increasing adoption of Western lifestyles in many parts of the world [3]. While PCa can occur at any age, the risk increases significantly above the age of 50. Consequently, PCa is considered an age-associated disease, with the majority of cases diagnosed in men aged 65 and older [4].

The prostate is a hormone-dependent organ, with its development and function largely regulated by androgens. The androgen receptor (AR) is a ligand-activated transcription factor and member of the nuclear hormone receptor superfamily that plays a critical role in both normal prostate development and the progression of PCa [5]. While a small amount of androgen is produced in the brain [6] and by the adrenal glands in the form of dehydroepiandrosterone (DHEA), a precursor that can be converted into testosterone, the majority of endogenous androgens are synthesized by Leydig cells in the testes [7]. AR-mediated signaling regulates prostate growth, differentiation, and is essential for the proliferation and survival of PCa cells [5, 8].

Advances in understanding the molecular mechanisms of PCa have led to the development of therapeutic strategies targeting the AR. Upon androgen binding, AR undergoes a conformational change, translocates into the nucleus, acts at the genomic level [1] by binding to androgen response elements (AREs) on DNA recruiting coregulators to initiate the transcription of target genes. AR target genes include genes encoding for prostate-specific antigen (PSA), a kallikrein family serine protease and an important diagnostic marker for PCa [9], for prostate-specific membrane antigen (PSMA) overexpressed in most PCa and exploited as an imaging marker and PSMA-targeted therapies, for transmembrane protease serine 2 (TMPRSS2) often genetically fused to the ERG gene or other ETS family members acting as oncogenes, and for the lncRNA PCA3 a FDA-approved diagnostic marker [10–12].

However, androgen deprivation therapy (ADT) with or without in combination with AR antagonists leads eventually to castration-resistance and therapy resistance. The molecular pathways that mediate a bypass for the inhibition of AR signaling are diverse, are often associated with enhanced AR expression and enhanced AR signaling. This indicates a high flexibility of tumor cells to respond to and bypass therapies leading to tumor evolution under selection of therapeutics. Nevertheless castration-resistance PCa cells still require AR signaling [13].

In addition to its genomic role, activated AR can also engage in rapid non-genomic signaling by interacting with various cytoplasmic effectors within minutes of androgen stimulation. These interactions may activate various signaling cascades that promote cell proliferation, survival, anti-apoptotic responses, and migration [14]. Notably, the PI3K-AKT-mTOR pathway is a well-established example of crosstalk between genomic and non-genomic AR signaling mechanisms [15, 16].

Circadian rhythms are autonomous anticipatory oscillators that widely exist in many organisms if not all [17]. The circadian rhythm is crucial for regulating numerous physiological processes and maintaining overall health. It plays a fundamental role in metabolism, sleep–wake cycles, hormone secretion, immune function, and cell cycle regulation [17–19]. Disruptions of circadian genes due to exogenous factors such as shift work, sleep disturbances, and nighttime light exposure are associated with an increased risk of various cancers, including breast, prostate, pancreatic, ovarian, and colorectal cancers [20–22].

In addition, circadian oscillations strongly influence pharmacokinetics and pharmacodynamics, prompting the field of chronopharmacology to optimize drug efficacy and minimize toxicity by timing treatments appropriately. Disrupting the cell cycle or the tumor’s circadian clock abolishes these rhythmic effects, highlighting the potential of leveraging circadian biology to optimize anticancer therapy timing [23, 24]. Endogenous factors such as single nucleotide polymorphisms of clock genes (SNPs) were identified by genome-wide studies and reported to be associated with various cancers, such as PCa [25]. Also, androgen signaling regulates the expression of clock genes [25], providing evidence of cancer risk and clock gene activity. Therefore, chronotherapy is an emerging field to analyze the timing of administration of therapeutics to coordinate the treatment with the biological clock in order to enhance therapeutic efficacy and reduce side effects.

Cellular senescence induces a cell cycle arrest that is elicited in response to different internal and external stressors [26]. Besides exiting from the cell cycle, senescent cells undergo many phenotypic alterations such as metabolic reprogramming, chromatin rearrangement known as senescence-associated heterochromatin foci (SAHF) and senescence-associated distention of satellites (SADS), epigenetic modifications, and autophagy modulation [27–30]. In addition, senescent cells produce and secrete a complex combination of factors, collectively referred to as the senescence-associated secretory phenotype (SASP), that mediate most of their non-cell-autonomous and microenvironmental effects [31]. The induction of premature senescence in cancer cells is increasingly being considered as a therapeutic strategy to limit tumor progression [32]. However, the induction of cellular senescence and the induction of SASP may promote via long-term medical administration, resistance to therapy-induced cellular senescence.

Accumulating evidence reveals that under certain conditions AR activation by SAL can paradoxically inhibit PCa proliferation. This finding has prompted clinical trials investigating bipolar androgen therapy (BAT), which alternates cycles of SAL with androgen deprivation. BAT is currently being evaluated in phase II trials, including TRANSFORMER, RESTORE, and COMBAT, with and without co-treatment of AR inhibitors or PARP inhibitors for patients with CRPC [33–36]. The use of SAL in BAT effectively inhibits PCa cell growth, indicating that SAL may activate a tumor-suppressive program [37]. It is also worth noting that BAT does not benefit all patients and preclinical and clinical data show that resistance mechanisms emerge over time [38, 39]. The limitation of BAT is that AR expression is required, and only AR-positive prostate cancers are responsive. A downregulation of AR, as an adaptive response, has been reported as a treatment limitation [38]. Interestingly, this effect could be reversed by treatment with AR antagonists that conversely induce an adaptive upregulation of AR [38].

Of note, both SAL and AR antagonists induce cellular senescence in CSPC and CRPC in adherent PCa cell lines, in PCa tumor spheroids and in mouse xenografts [40–43]. It emerges that the circadian clock is also a player in cancer biology including PCa. Intriguingly, both AR signaling and cellular senescence intersect with circadian regulators, suggesting a complex and understudied network that may govern PCa progression by SAL or AR antagonists. Despite significant advances in understanding each of these components individually, a comprehensive review of pathways of AR-mediated senescence and circadian regulation converging in PCa is currently lacking. This review aims to address that gap by critically evaluating the distinct mechanisms of AR-induced cellular senescence and exploring the role of circadian rhythm in PCa. It seeks to provide novel insights into PCa progression and therapeutic vulnerabilities, potentially paving the way for innovative treatment strategies, such as senescence-targeting therapies.

## Androgen receptor signaling in PCa

### AR structure and regulation by agonist and antagonists

The AR (NR3C4) is a member of the nuclear receptor superfamily and belongs to the steroid subfamily 3, group C, member 4.

The full-length AR is a protein of approximately 110 kDa consisting of mostly 920 amino acids (NM_000044.2) with a variable glutamine stretch in its amino-terminus. The AR protein contains four major functional domains: the N-terminal domain (NTD) spanning residues 1–555 encoded entirely by exon 1, the DNA binding domain (DBD) from residues 555 to 623, the hinge regions with residues 623 and 665, and the C-terminal ligand binding domain (LBD) from residues 665 to 920. The activation function-1 (AF-1), located within the NTD (residues 142–485) is constitutively active and includes two distinct transcription activation units Tau-1 and Tau-5 essential for the full activity of AR suggesting that the ligand binding domain inhibits the transcriptional activity of the N-terminus. Tau-1 contains the FQNLF nuclear receptor box, while Tau-5 contains the WHTLF motif with both mediating direct ligand-dependent intra- and intermolecular interactions between the NTD and LBD crucial for regulating some AR target genes [44]. The AR DBD is a cysteine-rich domain with two Zn-finger motifs that bind to the ARE motif of the genomic DNA. ARE mostly consists of two hexameric half-sites, being variable in sequence with the consensus motif 5’-AGAACA-3’ that is separated by a three-base-pair spacer (IR3) as inverted, direct repeats, or ARE half sites in close vicinity to other transcription factor binding sites [45]. ChIP-seq data suggest that AR is recruited to promoters, enhancers, and mostly to intronic regions of its many target genes exerting its effects through histone modifications and chromatin remodeling [9, 46].

The AR is widely expressed across various tissues influencing both normal physiology and disease states [9] and regulates numerous biological functions, including the development and maintenance of the prostate gland as well as modulating the cardiovascular, musculoskeletal, and immune systems [9]. AR plays a critical role in the growth and maintenance of prostate epithelial cells by trans-activating genes involved in cell growth, differentiation, and apoptosis [9, 47]. However, AR binding to chromatin sites is not always readily accessible. Pioneering factors such as FOXA1, studied extensively in PCa, facilitate chromatin remodeling by converting heterochromatin into euchromatin facilitating transcription factors like AR to bind to DNA [46]. AR binding to far distant sites from gene promoters can communicate with promoters through chromatin looping [46]. The large multiprotein mediator complex is a key player in chromatin looping. One of its components, MED1, interacts with AR while other subunits associate with RNA polymerase II and TATA box-binding proteins [46, 48]. Additionally, long non-coding RNAs, particularly enhancer lncRNAs (elncRNAs) and enhancer RNAs (eRNAs), reinforce and stabilize enhancer-promoter chromatin loops. These lncRNAs influence the recruitment of transcription factors and architectural proteins such as YY1, RAD21, and SMC3, with their expression levels correlating with enhanced enhancer-promoter interactions [49, 50].

AR ligands can be steroidal or non-steroidal molecules that bind to and modulate AR activity. Several steroidal natural agonists activate AR. Both testosterone (T) and its more potent metabolite, 5α-androstan-17β-ol-3-one (dihydrotestosterone, DHT) are produced via the enzyme 5α-reductase. DHT is more potent compared to testosterone binding with approximately 2 to 4 times higher affinity [51, 52]. The adrenal-derived steroid, 11-ketotestosterone (11KT), is notable for being one of the few endogenous steroids capable of activating AR at sub-nanomolar concentrations, similar to T and DHT. Importantly, 11KT is the predominant circulating active androgen in patients with castration-resistant PCa (CRPC) and is thus a potential driver of AR activation in CRPC [53, 54]. Several androgens secreted by endocrine glands including 4-androstene-3–17-dione (androstenedione), 5-androstene-3b,17b-diol (androstenediol), dehydroepiandrosterone sulfate (DHEAS), and DHEA, act as precursors in estrogen and testosterone biosynthesis [55, 56].

The growth of PCa is stimulated by testosterone, exemplifying the principle of hormone dependence, in which specific hormones play a crucial role in sustaining the survival of malignant cells. In contrast, the first successful demonstration that reducing hormonal status can induce cancer regression was achieved through the concept of hormone deprivation. This indicates that the AR acts as an oncogenic driver of PCa. In this treatment approach, malignant cells perish when their supporting hormones are withdrawn or their sources removed. This was evidenced by the striking and durable regression of advanced prostatic tumors following orchiectomy or the administration of estradiol benzoate or stilbestrol. Based on the studies examining the effects of castration, and androgen administration on serum phosphatase levels in metastatic prostate carcinoma, it was established that PCa is among the seven known types of malignancies responsive to hormonal modification [57].

Also, the beneficial use of AR antagonists supports the role of AR also as an oncogenic driver. Considering the growth inhibition by SAL, it suggests the AR has opposite functions dependent on the ligand and ligand concentrations. In contrast to AR agonists, several systemic AR antagonists have clinical significance for treating androgen-dependent disorders such as PCa. AR antagonists are also crucial tools for studying hormone action, resistance mechanisms, and androgen biology in disease and development [58]. The first-generation AR antagonists include flutamide and hydroxyflutamide being non-tissue-selective, but due to their metabolically susceptible nitro groups, they have very short half-lives and exhibit some liver toxicity [59, 60]. Bicalutamide, as another non-steroidal first-generation AR antagonist, is chemically a derivative of flutamide, binds reversibly to the AR-LBD and inhibits AR-mediated transactivation. Resistance against bicalutamide arises via AR overexpression or mutations in the ligand binding pocket such as the W741L mutation, which strikingly converts bicalutamide from an AR antagonist into a potent agonist [61, 62]. Enzalutamide (Enz, MDV3100) is the first FDA-approved second-generation AR antagonist for the treatment of patients with CRPC, exhibiting much higher AR-binding affinity compared to first-generation antagonists. It binds to the LBD of AR inhibiting androgen binding, reducing nuclear translocation, DNA binding, and co-activator recruitment [58]. Enz has been approved for both metastatic CRPC (mCRPC) and non-metastatic CRPC (nmCRPC). Importantly, Enz as other AR antagonists enhances the lifespan of PCa patients. Despite its widespread clinical use for CSPC and CRPC, Enz has a broad range of side effects including seizures and cardiovascular issues [58]. Apalutamide (ARN-509), another second-generation AR antagonist may present some fewer side effects compared to Enz [58]. The most recently developed AR antagonist is darolutamide (Dar, ODM-201), which shows a reduced risk of seizures. This may derive from its reduced ability to cross the blood–brain barrier [63]. Interestingly, Dar also inhibits the transcriptional activity of several AR mutants (F877L, F877L/T878A, and H875Y/T878A) that mediate resistance to Enz [64]. This suggests that different AR antagonists act in a distinct mode to inactivate the transactivation of AR.

Because drug resistance is observed by prolonged use of AR antagonists with the risk of a wide range of side effects, there is an urgent need for new AR antagonists and alternative therapeutic approaches. Some novel AR antagonists are currently in clinical trials such as proxalutamide and BMS-641988 [58]. Other AR antagonists targeting the amino-terminus of AR, such as masofaniten (EPI-7386) in combination with Enz, are currently under investigation [65]. Some other non-steroidal AR antagonists under pre-clinical investigation include the natural compound atraric acid (AA) [43] and the synthetic compound C28 [41]. Both have been studied in various CSPC and CRPC cell lines, as well as in CRPC xenograft mouse models. Both compounds inhibit AR, AR mutants that mediate resistance to first and second-generation AR antagonists, induce cellular senescence and suppress PCa cell growth. Similarly, pre-clinical evidence indicates inhibition of tumor growth by an AR degrader bavdegalutamide (ARV-110, a proteolysis targeting chimera, PROTAC) that especially in combination with abiraterone showed potent inhibition of growth in patient-derived xenograft and mouse model [66].

Besides AR-driven PCa, there exists an AR-indifferent subtype, in which tumor cells no longer rely on AR signaling for growth and survival. This state represents a key adaptive escape mechanism that emerges after prolonged treatment with potent AR pathway inhibitors such as abiraterone acetate or enzalutamide. Unlike most CRPC, often exhibiting high AR levels and remaining AR-driven through AR amplification, mutations, splice variants, or bypass pathways, AR-indifferent disease suppresses AR dependence.

AR-indifferent tumors exhibit lineage plasticity, losing their luminal epithelial identity and adopting neuroendocrine-like or stem cell-like phenotypes. Morphologically, they may still resemble adenocarcinomas but show markedly reduced AR activity, loss of luminal markers (e.g., PSA; Prostate Specific Antigen, PAP; Prostatic Acid Phosphatase), and clear signs of epigenetic and transcriptional reprogramming. Although AR protein may persist, its signaling output is minimal. This transition is driven by loss of the tumor suppressors RB1 and TP53, which together promote lineage plasticity and resistance to AR blockade through upregulation of SOX2, a pluripotency transcription factor that reprograms cells toward alternative fates. EZH2, a histone methyltransferase, reinforces this dedifferentiated state by silencing AR-regulated genes and remodeling chromatin. In addition, N-MYC amplification and AURKA overexpression cooperate to inhibit AR signaling and sustain tumor growth. AURKA stabilizes N-MYC, forming an oncogenic axis linked to therapy resistance. Clinically, AR-indifferent tumors are resistant to hormonal therapy, often produce low PSA levels, and are associated with visceral metastases (especially in the liver) and rapid progression despite castrate testosterone levels [67].

### Genomic and non-genomic AR signaling in PCa

**Fig. 1:**
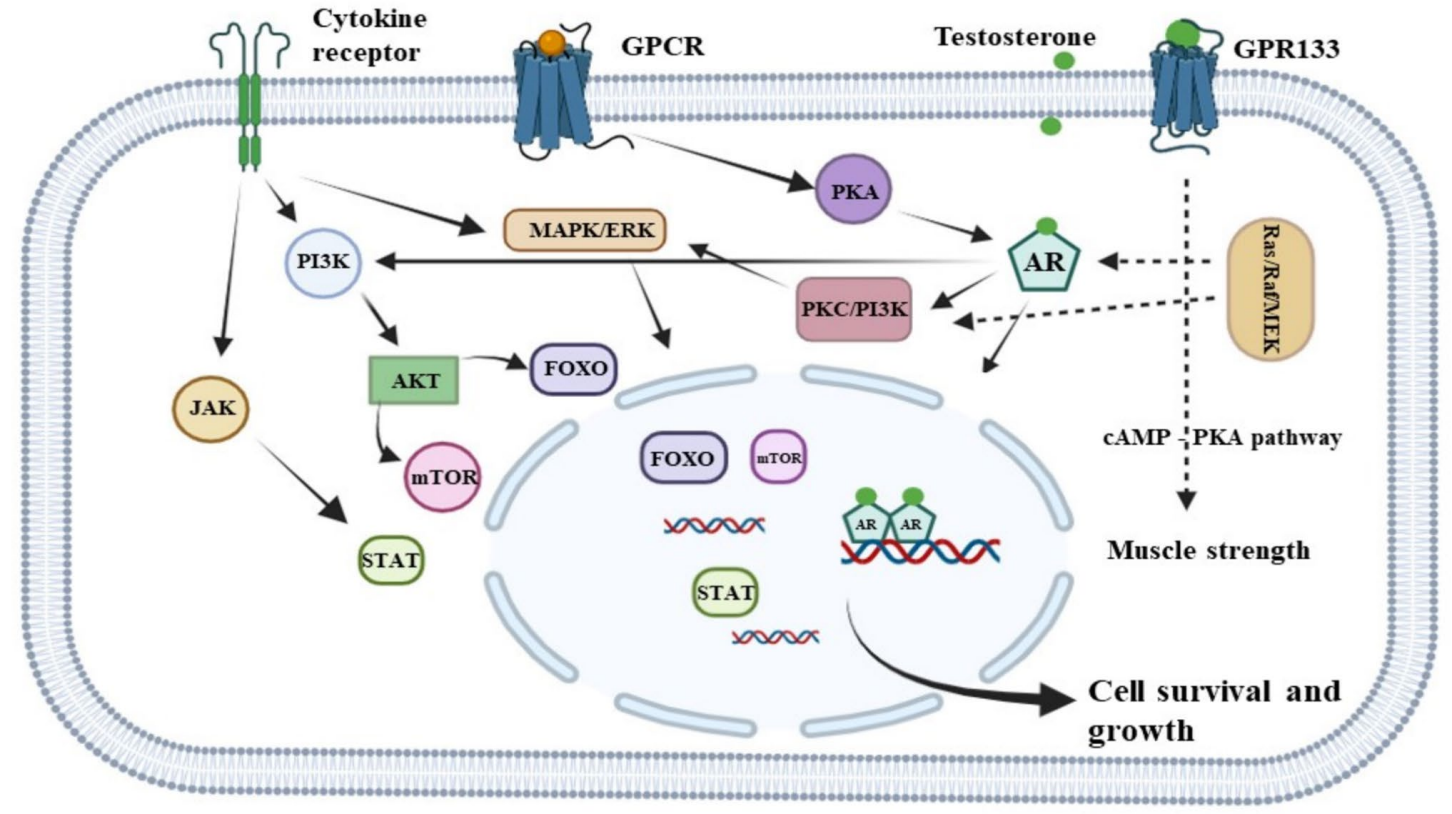
**AR genomic and non-genomic signaling pathways**. AR in the presence of androgen (testosterone at physiological level) through interaction with several factors and signaling axes including PI3K/AKT, JAK/STAT, Ras/Raf/MEK, and FOXO mediates cell survival and growth. Androgen can also activate GPR133, thereby increasing cAMP levels and enhancing muscle strength. Figure created with *BioRender.com* and modified

Androgen signaling, besides its role in PCa development and progression, is also implicated in other malignancies, including breast, bladder, liver, and kidney cancers [9]. Although the AR signaling pathway remains a cornerstone of PCa therapy. it was recently shown that DHT can activate GPR133, which represents an androgen membrane receptor that contributes to normal androgen physiology in muscle cells, thereby increasing intracellular cyclic AMP (cAMP) levels and enhancing muscle strength [68] (Fig. 1).

Of note, emerging evidence suggests that AR responds to different androgen concentrations in a dose-dependent biphasic manner. Whereas low androgen levels promote the growth of PCa cells, SAL treatment inhibits PCa growth. Mechanistically, it is suggested that the doses of the same androgen differentially affect the formation and abundance of AR monomers versus dimers or oligomers in cells [69]. These distinct forms of AR are associated with different biological outcomes; monomers are suggested to favor oncogenic and proliferative signaling, while dimers tend to drive tumor-suppressive transcriptional programs [69]. At physiological androgen concentrations, proliferative pathways such as E2F targets, G2M checkpoint regulators, and c-MYC targets are activated, presumably through monomeric AR signaling. In contrast, high androgen concentrations at supraphysiological levels (SAL) rather promote AR dimerization and classical genomic signaling, suppressing these same pathways [14, 69]. Thus, AR exerts dose-dependent and context-specific activities in PCa cells.

The role of androgens upon binding to AR is to induce a cascade of changes including conformational changes, posttranslational modifications, nuclear AR translocation, inducing a distinct protein- and RNA-interactome with the AR. The AR undergoes, e.g., serine phosphorylation at several sites (Ser81, Ser93, Ser641, Ser213, Ser506, and Ser650), which enhances its stability and transcriptional activity [70, 71]. These phosphorylation events are mediated by various kinases such as MAPK, ERK, p38, JNK, CDKs and AKT indicating that AR is part of many intracellular signaling networks linking posttranslational modifications to signal transduction and the cell cycle. The phosphorylation facilitates the recruitment of nuclear co-activators that mediate chromatin remodeling and induce chromatin modifications, thus enhancing AR-mediated genomic effects.

In contrast to genomic signaling, non-genomic AR signaling induces rapid cellular responses occurring within seconds to minutes that do not rely on transcription but can lead subsequently to changes in the transcriptome. At low androgen concentrations, AR remains rather predominantly monomeric and cytoplasmically localized, is able to activate mTOR signaling, to induce phosphorylation of Rb, leading to increased expression of E2F1 and FOXM1, key regulators of cell cycle progression [40, 69]. Interestingly, while both high and low androgen doses can activate mTOR, only low doses induce expression of c-MYC, a major oncogenic driver in PCa, whereas high doses suppress c-MYC expression [69]. These findings suggest that coordinated activation of mTOR and c-MYC by low-dose androgens underlies AR-driven PCa proliferation.

There is increasing evidence that non-genomic and genomic AR pathways intersect, potentially fine-tuning AR signaling in PCa cells. Mechanistically, non-genomic AR signaling often involves the MAPK/ERK, PI3K/AKT, Ras/Raf, and G-protein-coupled receptors (GPCRs) [72]. GPR133 is activated at lower androgen doses compared to the other GPCRs. Among the GPCRs, GPR56 and GPRC6A are androgen-responsive and play key roles in modulating AR activity. GPR56 activation rapidly stimulates protein kinase A (PKA) and Rho GTPase promoting AR signaling, cell proliferation, and migration as hallmarks of metastatic disease [73]. Notably, PKA activated by forskolin (FSK) facilitates androgen-independent AR activation, while PKA inhibition blocks AR nuclear translocation in LNCaP cells [74, 75].

GPRC6A responds to high testosterone levels by activating ERK and PI3K/AKT pathways, leading to mTOR activation and enhanced cell proliferation [76]. Even in the absence of androgens AR signaling can be driven by “outlaw” pathways involving cytoplasmic cascades such as PI3K-AKT, often triggered by growth factors or cytokines [14]. The PTEN tumor suppressor normally inhibits PI3K-AKT signaling. Therefore, loss of PTEN function results in constitutive AKT pathway activation, which promotes survival, growth, and proliferation via downstream targets such as mTOR, FOXO, BAD, TSC2, and GSK3 [72, 77].

The AR can also rapidly activate AKT through a non-genomic mechanism. This involves the interaction of AR with Src kinase, which subsequently stimulates PI3K signaling, leading to PIP3 production, membrane recruitment of AKT and its interaction with AR [78], leading to cross-phosphorylation of both AKT and AR [14]. While earlier studies identified their co-localization and interaction at membrane lipid rafts shortly after androgen treatment (~ 1 h), recent data suggest that prolonged androgen exposure (72 h) increases AKT phosphorylation and increases AR–AKT proximity in both cytoplasm and nucleus, possibly due to dislocation from lipid rafts [79, 80].

Cytokine signaling also crosstalks with AR pathways. For instance, interleukin-6 (IL-6) activates JAK/STAT3 and MAPK pathways, which interact with AR signaling [81]. Similarly, the Ras-Raf-MEK-MAPK-ERK axis is engaged downstream of AR activation, with dominant-negative constructs of Raf-1 and PI3K-p85 shown to block DHT-induced ERK activation. This underscores the multifaceted interaction between AR, Src, PI3K, AKT, mTOR, PKC, and MAPK pathways in driving PCa progression [77].

## Cellular senescence in cancer: a double-edged sword

### Cellular senescence and its hallmarks

The induction of cancer cell senescence may represent a strategy for anti-cancer treatment [82]. Cellular senescence was first described by Hayflick and Moorhead as an irreversible cessation of cell division after a finite number of replications, a phenomenon now known as the Hayflick limit [83]. The widening definition of cellular senescence refers to a cellular state in which cells cease replication in response to a wide variety of intrinsic and extrinsic stimuli, including telomere shortening, therapy-induced senescence, oxidative stress, DNA damage, and circadian rhythm dysregulation [84, 85].

Several features and alterations are associated with cellular senescence in human cells, including morphological changes, SASP, SAHF, and SADS formation, overexpression of cell cycle inhibitors, elevated levels of senescence-associated β-galactosidase (SA-β-Gal) activity, and epigenetic modifications. The process of cellular senescence can be divided into two phases: the initial phase, which includes changes such as chromatin rearrangement and cell cycle arrest, and the complementary phase, during which senescent cells secrete factors that affect neighboring cells, known as the SASP. In this section, each feature is briefly described.

Abnormal cytoplasmic DNA fragments in senescent cells, often resulting from mitochondrial dysfunction and the reduced expression of Lamin B1, destabilize the nuclear structure that can leak into the cytosol. These fragments, such as cDNA derived from the retrotransposon LINE-1, act as ligands for the DNA sensor cGAS. This activation leads to the production of cyclic dinucleotides recognized by STING, ultimately triggering type I interferon-producing pathways [86, 87].

Nuclear chromatin is broadly categorized into euchromatin and heterochromatin. These two forms of chromatin exhibit distinct post-translational histone modifications and chromatin-binding proteins [88]. During the induction of cellular senescence in human cells, chromatin undergoes profound structural alterations in senescent cells leading to the formation of SAHF, which is characterized by heterochromatin protein 1 (HP1), methylated histone 3 lysine 9 (H3K9), and the presence of histone variants including macroH2As [89]. Di- or tri-methylation of H3K9 (H3K9me2/3) is an epigenetic marker of heterochromatin. HP1 specifically recognizes H3K9me2/3, contributing to transcriptional repression and the spread of heterochromatin. HP1 also plays a role in regulating the stability of enzymes involved in H3K9 methylation dynamics [90]. Furthermore, inhibition of the mTORC1/p70S6K pathway has been shown to promote heterochromatin organization by increasing the expression of H3K9me3 and HP1γ, thereby maintaining heterochromatin integrity and potentially protecting DNA from further damage during stress-induced senescence [91].

In senescent cell nuclei, SAHF forms around endogenous persistent DNA double-strand break (DSB) sites and suppresses the entire DSB response including the phosphorylation of H2AX (γ-H2AX) and recruitment of other repair proteins [89]. SAHF is enriched with macroH2A isoforms, although not all SAHF contains macroH2A foci [92]. MacroH2As consist of an N-terminal H2A-like domain and a large C-terminal macro domain. There are three isoforms, macroH2A1.1, macroH2A1.2 (splice variants), and macroH2A2 [92]. De novo deposition of macroH2A into chromatin is closely associated with activation of the HIRA/ASF1a chromatin-remodeling pathway. HIRA interacts with ASF1a, a histone chaperone, in a critical step toward SAHF formation. ASF1a may further contribute to macroH2A incorporation by facilitating chromatin disassembly and allowing accumulation of macroH2A by additional factors [92, 93].

Another global chromatin change is SADS, first described by Swanson et al., that refers to the dramatic unfolding of satellite DNA that occurs early in senescence. Unlike SAHF, which is not observed in all senescent cells, SADS is more universally present. Cells can exhibit SADS without SAHF, but seemingly not vice versa. However, SADS is generally not observed in immortalized or transformed cancer cell lines, although they have been reported in benign prostate tumors composed of senescent cells [28, 94]. The molecular mechanisms and functions of SADS remain unclear. SADS formation may be linked to hypomethylation and the expression of pericentric satellite DNA during senescence [29]. Additionally, depletion of histone deacetylase 3 (HDAC3), an epigenetic regulator in bone marrow-derived osteoprogenitor cells, has been shown to promote SADS formation and early senescence [95].

Morphological characteristics of senescent cells may include a flattened appearance, enlarged nuclei, sometimes multinucleated, extensive vacuolization, and reduced saturation density at the plateau phase of cell growth [82, 96]. Furthermore, disruption of nuclear envelope integrity has been observed in senescent cells due to reduced Lamin B1 expression [96]. Most of the non-autonomous effects of senescent cells are attributed to the SASP [97].

Cancer cell senescence not only prevents the proliferation of malignant cells but also communicates with neighboring cells and alters the tissue microenvironment through SASP. SASP is highly heterogeneous in cancer cells and can influence the tumor microenvironment in diverse ways. On one hand it is beneficial and may induce cellular senescence of neighbor cancer cells; on the other hand, it may promote malignant cell proliferation. This heterogeneity may also affect the morphology and behavior of senescent cancer cells in the microenvironment [82, 87, 97].

Jochems et al. analyzed the transcriptome and senolytic responses induced by Navitolax (ABT-263), an orally bioavailable anti-apoptotic Bcl-2 inhibitor, in a panel of 13 cancer cell lines rendered senescent and found that the composition of the SASP is more influenced by the cell of origin rather than by the senescence trigger. IL-6 and IL-8 (CXCL8) are well-known classical SASP factors, but even their expression and levels vary depending on the cancer type and type of senescence inducer. IL-6 expression tends to increase primarily in lung and breast cancer cell lines, especially those with high baseline IL-6 expression, whereas CXCL8 expression shows a less consistent pattern increasing in some cell lines but not in others [98]. On the other hand it has been reported that the type of senescence inducer exhibits a distinct SASP [99] Key pathways that regulate SASP includes NF-κB, C/EBPβ, and cGAS-STING signaling [100]. Biomarkers detecting SASP may be a helpful tool in diagnostics. Using senoylitcs, that kill senescent cells, or senomorphics that ideally suppress pro-inflammatory SASP components but permit tissue regeneration may be used as therapies, but off-targets should be reduced to lower side-effects.

Senescence-associated β-galactosidase (SA-β-Gal) is a lysosomal hydrolase active at pH 6.0 and the classic and gold-standard biomarker to detect cellular senescence. This enzyme cleaves terminal β-D-galactose residues from substrates such as lactose, keratin sulfates, and sphingolipids. SA-β-Gal activity can be detected via X-gal staining, where cells containing active enzyme produce a blue precipitate upon cleavage of X-gal (5-bromo-4-chloro-3-indolyl β-D-galactopyranoside) allowing visual identification [101]. While this assay is restricted to fixed cells, a biocompatible and injectable organic nanoprobe known as NanoJagg has been developed. NanoJaggs are selectively taken up by senescent cells, accumulate in lysosomes, and can be detected via imaging or photoacoustic tomography. *In vitro*, *ex vivo*, and *in vivo* studies have demonstrated that NanoJaggs are promising probes with potential for clinical translation for cell senescence detection [102].

### Inducer of cellular senescence in PCa

Cellular senescence in PCa is now recognized as a complex process with both beneficial and detrimental effects. The role of the AR as a key factor in PCa cells and its significance in mediating cellular senescence is becoming increasingly understood. Interestingly, both AR agonists and antagonists, despite their opposing effects on AR transcriptional activity, have been shown to induce cellular senescence in PCa cells [40, 43, 79].

**Fig. 2:**
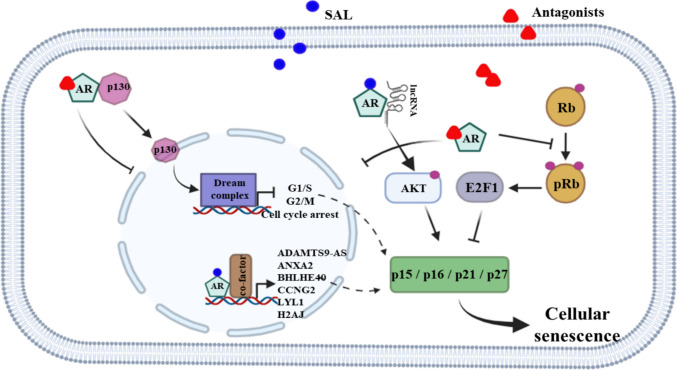
**Cellular senescence regulation in PCa via AR agonists and antagonists. **SAL induces cellular senescence through multiple AR-associated pathways. Key mechanisms include activation of AR-AKT signaling, repression of the oncogenic lncRNA MIR503HG influencing AKT-p70S6K and pRb-E2F1 pathways, induction of ANXA2, activation of the circadian factor BHLHE40, which drives the atypical tumor suppressive CCNG2, p15^INK4b^, p21^Waf1/Cip1^, and LYL1-p27^Kip1^, and up regulation via the histone variant H2AJ contributing to SAHF formation. Conversely, AR antagonists (Bicalutamide, ENZ, Dar, atraric acid, and C28) also induce senescence through p16^INK4a^ upregulation, Rb hypo-phosphorylation, inhibition of AR nuclear translocation, DREAM complex activation. Collectively, both AR agonists and antagonists engage distinct but overlapping pathways to promote cellular senescence in PCa. Figure created with *BioRender.com* and modified

In this section, we focus on the current understanding of AR-regulated cellular senescence in PCa (Fig. 2).

#### AR-agonist mediated cellular senescence

AR agonists at SAL, used in BAT, induce cellular senescence leading to PCa growth inhibition [40, 41, 103]. Several AR-associated pathways are known to be involved in regulating cellular senescence.

The phosphorylation of AKT was shown to be a mediator of SAL-induced cellular senescence. It has been shown that AKT inhibition attenuates SAL-induced cellular senescence, suggesting the involvement of the AR–AKT signaling pathway in senescence regulation [40].

Being in line with tumor suppressive activity induced by SAL, some tumor suppressors are known to be within the AR signaling and SAL-induced cellular senescence. Both Inhibitor of Growth factors, ING1 and ING2, belonging to a family of type II tumor suppressors are interacting partners of the AR and mediate cellular senescence. ING1b and ING2 act as transcriptional corepressors of the AR and share at the transcriptional level common differentially expressed genes. One key factor within the AR-ING pathway that inhibits replicative senescence is the human catalytic subunit of the telomerase TERT, which has in addition to telomeric function also non-telomeric oncogenic activity [104]. Interestingly, hTERT is a negatively regulated AR target gene at SAL. Mechanistically, the tumor suppressors ING1b and ING2 mediate AR-induced hTERT repression as corepressors supporting the notion that SAL has tumor suppressive activity. Interestingly these findings also provide some mechanistic evidence of an underlying mechanism of how the SAL-activated AR can repress target genes. Additionally, ING1b induces BCL-2, a well-known anti-apoptotic protein, which also modulates entry into cellular senescence through the regulation of p16^INK4a^, p27^Kip1^, and p21^Waf1/Cip1^ both dependently and independently of AR via crosstalk with ING2 in various PCa cells [105, 106].Annexin2 (ANXA2), a member of the Annexin A family acts as a tumor suppressor in PCa with higher expression significantly associated with improved overall survival in PCa patients. Mechanistically, SAL enhances AR recruitment to the ANXA2 gene locus and increases its expression indicating that ANXA2 is a direct and positively regulated AR target. Knockdown of ANXA2 reduces SAL-induced cellular senescence [107] suggesting that ANXA2 is part of the tumor suppressive program of SAL.

lncRNAs also play an important role in mediating SAL-induced cellular senescence. A directly AR-repressed gene is the lncRNA MIR503HG, which exhibits increased expression in metastatic PCa further supporting the notion that SAL induces a suppressive tumor pathway. The lncRNA MIR503HG has been indicated to act as an oncogenic lncRNA and shown to promote PCa cell proliferation and suppress SAL-induced cellular senescence partly via miR-424-5p [108]. On the other hand, SAL upregulates the expression of the pro-survival lncRNA SAT1 in PCa cells which, through increased phosphorylation of AKT at S473 and upregulation of the p15^INK4b^ cell cycle inhibitor contributes to the induction of cellular senescence [79]. These findings underscore also the importance of noncoding RNAs in regulating senescence in PCa within AR signaling. In support of the role of lncRNAs in regulating cellular senescence, another study reported that SAL differentially regulates the lncRNAs ADAMTS9-AS2 and PART1. The expression of ADAMTS9-AS2, suggested to induce tumor-suppressive pathways, is induced by SAL and mediates SAL-induced cellular senescence, whereas the expression of PART1 is repressed by SAL and inhibits SAL-mediated cellular senescence in PCa cells [109].

The role of histone variants in cellular senescence remains less understood. The most well-studied variant H2AX marks sites of DNA damage and initiates the DNA damage response serving as an indicator of genomic instability during aging and senescence [110]. Recently, it was demonstrated that the H2AJ variant is a direct AR target gene and a downstream factor in AR signaling. H2AJ partially regulates SAHF formation, SAL-induced cellular senescence and contributes to PCa cell growth [111].

Moreover, the transcription factor LYL1, as part of the AR-BHLHE40/LYL1-p27^Kip1^ axis, was also found to regulate SAL-mediated cellular senescence in PCa cells. This axis functions through three interconnected feedback loops, with the cell cycle inhibitor p27^Kip1^ serving as a critical mediator, acting downstream of the BHLHE40–LYL1 interplay [112].

Collectively, these findings illustrate how SAL influences the complexity of AR signaling to suppress PCa cell growth through the induction of cellular senescence. Also, it may highlight the presence of compensatory and bypass mechanisms as well as feedback loops, both positive and negative, within the AR signaling axes that may modulate senescence.

#### AR antagonists mediated cellular senescence

Various AR antagonists also induce cellular senescence in PCa. This includes clinically used first- and second- generation AR antagonists such as bicalutamide, Enz and Dar [57, 113]. Also, the first identified natural AR antagonist, atraric acid, identified in barks of *Pygeum africanum* that has been used since generations to treat prostatic issues [113] induces cellular senescence. AA inhibits AR-mediated transactivation also of various AR mutants known to mediate resistance to AR antagonists and suppresses the proliferation of AR-expressing PCa cell lines. AA reduces nuclear translocation and chromatin recruitment of AR and leads to induction of cellular senescence [114]. C28, a derivative of methyl anthranilate, harbors only one benzene ring similar to AA. C28 has been shown to inhibit both wild-type AR and AR mutants that mediate resistance to clinically used AR antagonists such as hydroxyflutamide, Bic, Enz [41, 115]. Mechanistically, C28 inhibits AR translocation presumably by reducing phosphorylation of both AR at serine 81 and HSP27. Both events are required for full AR nuclear translocation. C28 enhances the interaction between AR and the pocket domain protein p130. Notably, increased p130 levels were also observed upon SAL treatment, leading to activation of the DREAM complex signaling and the induction of cellular senescence. This suggests that both AR agonists and antagonists may have distinct and shared pathways for the induction of cellular senescence and growth repression [41].

Enz and Dar, members of the second-generation AR antagonists, induce cellular senescence in PCa, competitively block the AR-androgen interaction and reduce AR translocation to the nucleus, thereby weakening its recruitment to chromatin [85, 116, 117].

### Targeting senescent cells using senolytic compounds

Since within the tumor microenvironment SASP may promote therapy resistance, senolytic compounds may be useful as combination treatments. Senolytics selectively eliminate senescent cells by exploiting the dependence of tumor cells on specific survival pathways induced by treatment [118, 119].

The combination of the tyrosine kinase inhibitor dasatinib and the flavonoid quercetin was among the first to demonstrate a reduction in senescent cell burden and improvement in physiological function in aged mice, with subsequent advancement into pilot human studies [120]. Fisetin, a plant-derived flavonol with senolytic activity, has an attractive safety profile compared with certain chemotherapeutics and is currently being evaluated in multiple early-phase clinical trials targeting age-related frailty and mobility outcomes [121]. Navitoclax (ABT-263), a BCL-2/BCL-XL family inhibitor originally developed as an anti-cancer drug, selectively eliminates senescent cells dependent on BCL-2 family survival signaling; however its use is limited by tissue toxicity [122]. Peptide-based approaches, such as the FOXO4-DRI peptide, which disrupts protein–protein interactions essential for senescent cell survival have shown promising preclinical efficacy [119]. Additionally, HSP90 inhibitors and p53/MDM2 interaction inhibitors have demonstrated senolytic activity in experimental models. Importantly, although several senolytic candidates have progressed to clinical trials some have failed to meet primary efficacy endpoints underscoring the translational challenges in this field [123, 124].

## The circadian clock: molecular architecture and roles in cancer and cellular senescence

Circadian rhythm comprises the core and peripheral clocks. The core and peripheral clocks exhibit a synchronous circadian rhythm [19]. The transcription-translation feedback loop (TTFL) is the molecular basis of circadian rhythms in organisms [19]. The positive stimulus factor TTFL comprises the factor circadian locomotor output cycles kaput (CLOCK) and brain and muscle ARNT-like protein 1 (BMAL1), while the negative stimuli for TTFL are cryptochrome (CRY1/2) and period (PER1/2/3) factors [19, 125, 126]. Therefore, two different complexes of TTFL regulators, the CLOCK-BMAL1 transcriptional activators and the CRY-PER transcriptional inhibitors, whose interplay is the basis of the main concept of circadian rhythm.

### Core and peripheral clock genes

**Fig. 3:**
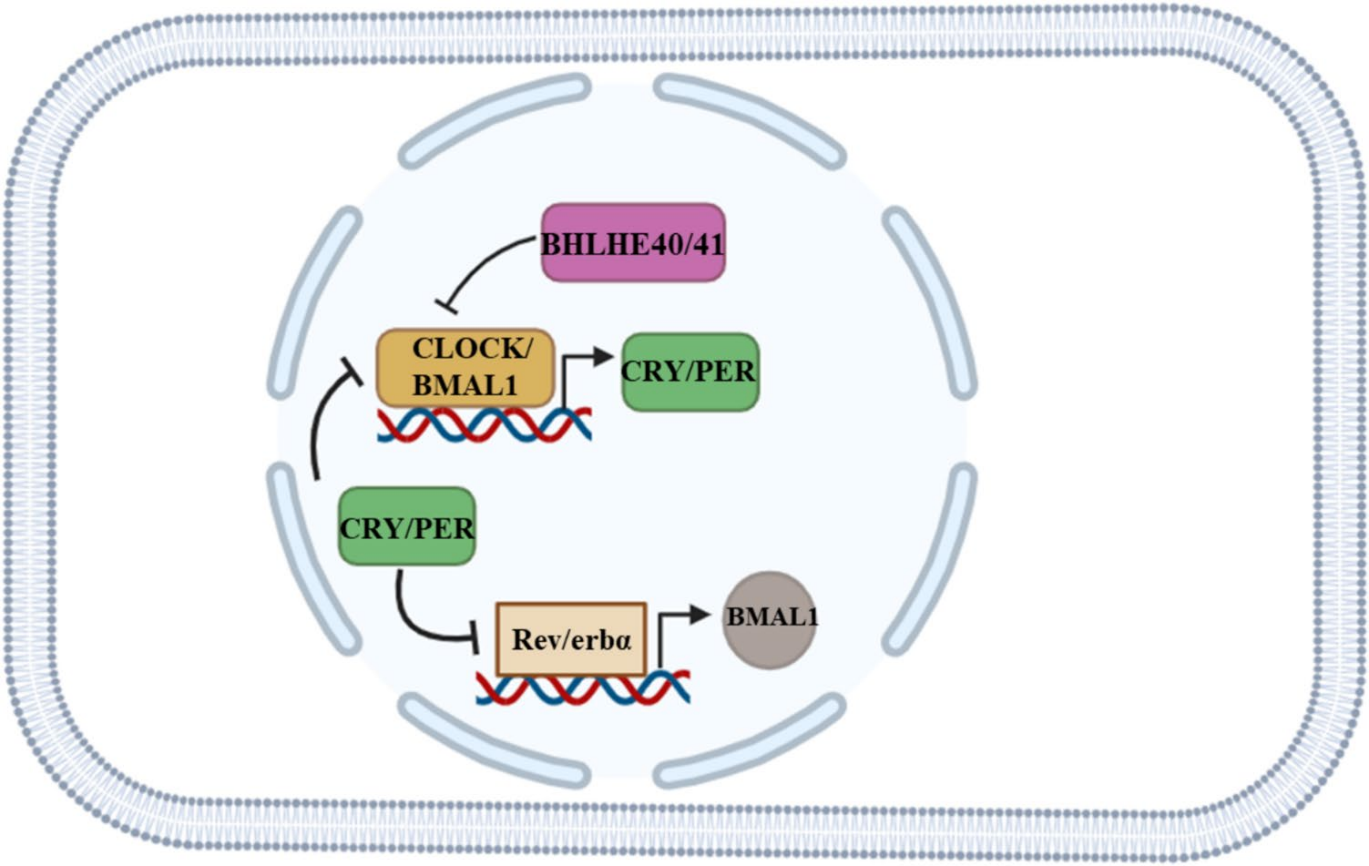
**Core and peripheral circadian clock pathways**. Core clock genes (CLOCK, BMAL1, CRY and PER) and peripheral clock genes such as REV-ERBα, BHLHE40, and BHLHE41 through several transcriptional and translational feedback loops, control biological processes such as endocrine hormone secretion. Figure created with *BioRender.com* and modified

The circadian clock derived from the Latin words "circa" (about) and "diem" (day), is a natural, internal time-keeping system that regulates biological processes including behavior, physiology, and metabolism such as sleep–wake cycles and hormone release in a roughly 24-h cycle. Core clock genes are the key regulatory components of the molecular circadian clock that generate and maintain rhythms [127, 128] (Fig. 3).

The primary circadian genes are CLOCK, BMAL1, PER, and CRY. The first identified clock component gene was PER, which was discovered in 1971 in *Drosophila* [129]. The Clock gene was first discovered in the 1990 s in mice. The encoded protein belongs to the basic helix-loop-helix (bHLH) protein family [130]. Another circadian factor with a critical role in circadian regulation is BMAL1 [131]. The encoded protein dimerizes with the CLOCK protein. CRY is a blue light-responsive flavoprotein photopigment related to photolyases, so named because its function was cryptic at the time of its initial identification. In mammals, two cryptochrome genes, CRY1 and CRY2, have been identified as being highly expressed in the ganglion cells and inner nuclear layer of the retina [132]. These genes play a crucial role in the feedback regulatory loops between circadian genes that generate circadian rhythms. CLOCK and BMAL1 are transcription factors that act as positive regulators in the promoters of target genes, including PER and CRY, initiating their transcription during the day. Conversely, PER and CRY proteins translocate to the nucleus and inhibit the activity of the CLOCK–BMAL1 complex. This negative transcriptional–translational feedback loop among circadian factors results in rhythmic gene expression over a 24 h cycle. Disruption of this system can affect the expression of clock genes, leading to sleep disorders, metabolic syndromes, and dysregulated cancer-related genes [133, 134]. Dozens of other candidate genes that play additional roles in the circadian gene network have been identified, which comprise the peripheral circadian clock regulators, including the feedback loop involving the members of nuclear hormone receptors REV-ERBα/RORα, and the transcription factors BHLHE40/BHLHE41 [135, 136]. The participation of nuclear hormone receptors as clock regulators suggests some hormonal regulation of circadian rhythm.

Recently, clock genes have functionally been associated with AR signaling and SAL-mediated cellular senescence. The circadian clock factor BHLHE40 is upregulated in PCa cells following SAL treatment. Through AR signaling, BHLHE40 upregulates the atypical cyclin gene CCNG2 along with p15^INK4b^ and p21^Waf1/Cip1^, to mediate cellular senescence [42, 112]. These findings suggest that circadian clock genes may have regulatory roles in cellular senescence beyond their function in circadian rhythm.

REV-ERBα, (NR1D1), a member of the nuclear hormone receptor superfamily completes the PER-CRY/CLOCK-BMAL1 rhythm loop (Fig. 3). The promoter region of NR1D1 contains three E-box DNA motifs known to be bound by HLH transcription factors. The NR1D1 gene is transcriptionally positively regulated by CLOCK and BMAL1 [137]. While its transcription is negatively regulated by PER and CRY, the REV-ERBα protein plays a crucial role in driving the circadian oscillation of BMAL1 transcription [137]. The FBXL3 protein encoded by another circadian gene, FBXL3, leads to degradation of CRY1 [138]. NPAS2 (neuronal PAS family member 2) shares the closest homology with CLOCK and in the absence of functioning CLOCK, NPAS2 appears to be able to partially compensate [139]. Like other clock genes, BHLHE40 and −41, also known as DEC1 and 2 (Differentiated Embryo Chondrocyte), respectively, are basic helix-loop-helix transcription factors that bind to DNA. These two transcription factors form heterodimers and suppress PER transactivation by interfering with the CLOCK-BMAL1 complex [140, 141]. BHLHE40 is also involved in an auto-feedback loop with CLOCK-BMAL1 to regulate its own expression [142].

### Circadian clock genes in cancer

There is growing evidence linking circadian clock genes to cancer [143, 144]. Disturbances in circadian rhythm have been associated with an increased risk of developing cancer [18, 145]. Disruption of circadian genes due to factors such as shift work, sleep disturbances, and nighttime light exposure is associated with an increased risk of various cancers, including breast, prostate, pancreatic, ovarian, and colorectal [20–22]. Disruption of the circadian clock is emerging to play an important role in cancer-associated metabolic reprogramming, driving hallmark alterations such as enhanced aerobic glycolysis (Warburg effect), increased glutamine oxidation, elevated lipogenesis, and increased nucleotide synthesis in cancer cells [145].

In line with this, single nucleotide polymorphisms (SNPs) in many circadian clock genes have also emerged as important factors influencing PCa risk, progression, and treatment response [146] further supporting an important role of dysregulated clock genes in the risk of PCa. The rs7950226 and rs142435152 SNPs in the BMAL1 gene locus are linked to an increased risk or overall susceptibility to PCa [147, 148]. Several SNPs in the CRY1 gene locus, including rs12315175, rs10778534, rs7297614, and rs1921126, are also associated with an increased risk of PCa or with fatal disease outcomes [147, 149, 150]. The rs1401417 variant in the CRY2 locus may present an elevated risk for PCa particularly in men with higher insulin resistance, whereas rs2292912 is associated with a decreased risk of PCa [147, 151]. In addition, PER1 (rs885747, rs2289591), PER2 (rs7602358), and PER3 (rs1012477, rs228697) SNPs are implicated in PCa risk [147]. Collectively, these findings demonstrate that polymorphisms in circadian genes play a significant role in influencing PCa susceptibility, progression, and mortality [146].

Another link between clock genes and PCa risk derives from an analysis of PCa stemness and therapy resistance. Circadian clock genes also control PCa progression and therapeutic resistance, including prostate cancer stem cells (PCSCs). Notably, BMAL1 is strongly enriched in post-enzalutamide-treated PCa tissues and is essential for sustaining the growth of enzalutamide-resistant PCa cells, highlighting its potential as a therapeutic target in androgen-independent PCa. In parallel, PER3 expression is significantly reduced in ALDH^hi^CD44^+^ PCSCs abundant in CRPC. PER3 functions as a suppressor of stemness in these ALDH^hi^CD44^+^ cells by modulating the WNT/β-catenin signaling pathway, thereby pointing toward circadian rhythm disruption as a promising therapeutic avenue in CRPC. In PCa, CRY1 serves as a tumor-promoting factor, with its expression specifically induced by androgens through direct AR binding. The elevated CRY1 protein is subsequently stabilized following DNA damage, enabling it to regulate transcriptional programs critical for cell cycle progression, including the G2/M transition, while also coordinating homologous recombination (HR) by directly binding to and activating key HR repair factors such as ATM and RAD51 in a sequential manner, thereby ensuring efficient DNA repair and supporting cancer cell survival beside its canonical circadian function [152].

Of note, a circadian rhythm-related signature was developed comprising seven genes (FBXL22, MTA1, TP53, RORC, DRD4, PPARGC1A, ZFHX3) that strongly predicted relapse-free survival in prostate adenocarcinoma, outperforming standard clinical indicators. Combined with clinical T stage, it yielded a 5-year very high AUC of 0.944 and was associated with higher tumor mutation burden, microsatellite instability, and immune checkpoint expression suggesting potential for predicting immunotherapy response. According to their analyses this signature was linked to mitochondrial metabolism, oxidative phosphorylation, and cancer invasion-related pathways [153].

Chronotherapy optimizes cancer treatment by aligning drug administration with the body's circadian rhythms to enhance efficacy and minimize side effects and there are three primary chronotherapeutic approaches that have been employed in a combinatory manner for cancer treatment.

I. Training the clock, this involves interventions aimed at enhancing or maintaining a stable circadian rhythm in feeding-fasting, sleep–wake, or light–dark cycles.
II. Drugging the clock, this approach utilizes small-molecule agents that specifically target the circadian clock.
III. Clocking the drugs, this strategy focuses on optimizing the timing of drug administration to enhance efficacy and minimize adverse side effects [154].

In the context of training circadian clocks, light therapy has emerged as a promising chronotherapeutic tool to help reduce cancer progression. Several studies using a human cancer cell xenograft model have demonstrated that exposure to blue light during the day inhibits the nighttime circadian regulation of melatonin and tumor growth of prostate, liver, and breast cancers [155, 156].

An interesting new aspect to regulate circadian rhythm emerged by analyzing nutritional interventions, such as caloric restriction (CR). Interestingly, this is increasingly recognized for its potential to enhance circadian rhythm [157]. In addition to its anti-aging effects, CR exhibits anti-cancer properties by slowing tumor progression, promoting cancer cell death, and increasing the effectiveness of chemotherapy and radiotherapy [158]. In the realm of drugging circadian clocks, the identification of small molecules that can modulate circadian rhythm by targeting primary or secondary clock proteins has broadened the treatment options and windows for patients with various clock-related disorders [159]. For example, casein kinases are recognized as pro-oncogenic proteins and are emerging as promising therapeutic targets in cancer treatment. A range of inhibitors targeting casein kinases 1δ and 1ε (CK1δ/ε) have demonstrated antitumor effects in the treatment of breast cancer [160]. Additionally, various ROR isoforms and positive transcriptional regulators of BMAL1 expression have been targeted for cancer treatment. Inhibiting RORγ has shown potent antitumor activity across multiple cancer types, including CRPC [161]. With well-documented evidence that circadian clocks influence the absorption, distribution, metabolism, and elimination of drugs, the timing of medication administration becomes a crucial factor in disease treatment, particularly in chemotherapy [162]. Early studies with patients having advanced ovarian cancer indicated that administering doxorubicin in the morning (e.g., at 6 a.m.) and cisplatin in the evening (e.g., at 6 p.m., either before or after doxorubicin) resulted in fewer complications and reduced renal toxicity [163].

In fact, androgen production, particularly testosterone, is regulated by the circadian rhythm with levels exhibiting diurnal variation that is tightly controlled by the hypothalamic-pituitary–gonadal axis. Circadian regulation of androgen production begins with the master clock in the suprachiasmatic nucleus (SCN), where core clock genes such as BMAL1 and CLOCK drive rhythmic output signals that control the pulsatile release of GnRH from the hypothalamus. GnRH in turn, stimulates LH secretion from the anterior pituitary, which activates cAMP/PKA signaling in testicular Leydig cells. In these cells, peripheral clocks composed of PER1/2, CRY1/2, and REV-ERBα modulate the circadian transcription of key steroidogenic enzymes including StAR, CYP11A1, CYP17A1, and HSD3B, thereby orchestrating the daily rhythm of testosterone biosynthesis [164, 165].

### Circadian clock genes controlling cellular senescence

Over the past decade, a substantial body of evidence has accumulated indicating that disruption of the circadian clock is associated with age-related diseases and premature aging. Aging has been recognized as a significant risk factor that contributes to alterations in the circadian clock. At the molecular and cellular levels, there is significant crosstalk between the circadian clock machinery and various processes, including the cell cycle, DNA repair, apoptosis, senescence, autophagy, and other oncogenic and immune pathways [162]. BMAL1, one of the primary circadian genes, its expression decreases in the natural aging process and premature cellular senescence [166]. In summary, the protein complex mammalian target of rapamycin complex 1 (mTORC1) regulates the circadian clock by phosphorylating BMAL1 through its effector kinase S6K1. Additionally, mTORC1 activity is influenced by circadian clock dampening during senescence [167]. Accumulative data in other studies revealed that NAD^+^-dependent deacetylases SIRT1 and SIRT6 rhythmically deacetylate the acetylated histone H3 on circadian clock gene promoters, thereby producing rhythmic gene expression [168]. SIRT1 also rhythmically deacetylates BMAL1, which plays a key role in regulating the recruitment of CRY to the CLOCK-BMAL1 complex [169], thus linking metabolism and circadian rhythm by NAD^+^ as the cofactor for sirtuins. Furthermore, SIRT1 influences PER2 by modulating its protein stability and subcellular localization [170]. PARP1, also an NAD^+^-dependent enzyme, rhythmically binds to and poly (ADP-ribosyl)ates CLOCK, thereby modulating the binding affinity of the CLOCK-BMAL1 complex with PER-CRY repressors [171]. Therefore, reduced levels of NAD^+^ in senescent cells may contribute to the impaired circadian clock function observed in these cells [84]. Since the core circadian factors CLOCK and BMAL1 form a heterodimer that cooperatively promotes Nonhomologous End Joining (NHEJ) and Homologous Recombination (HR) DNA repair of double-strand breaks by stabilizing genome integrity and thereby antagonizing cellular senescence [172] it suggests a link between circadian rhythm, ligand-induced cellular senescence and DNA repair.

Mechanistically, a study by Jia et al. demonstrated that the accumulation of CRY1, a core clock gene, in bladder cancer cells protects them from therapy-induced cellular senescence. The KD of CRY1 restores the ability of paclitaxel to induce cellular senescence in these cancer cells [173]. Overexpression of BHLHE40 induces G1 arrest and promotes senescence in esophageal squamous cell carcinoma in a p21^Waf1/Cip1^-independent manner [174]. Also in PCa it was shown that BHLHE40 mediates SAL-induced cellular senescence by regulating atypical CCNG2 and LYL1 another HLH transcription factor [42, 112].

## Conclusion

PCa remains a major global health concern, particularly in aging populations, and its progression is intricately linked to AR signaling, hormone regulation, and cellular senescence. In this review, we explored the interconnected biological systems underlying PCa progression, focusing on how AR signaling, the circadian clock, and cellular senescence converge to influence cellular senescence in cancer cells. Together, these three systems form a dynamic and underexplored network with significant implications for disease progression and therapy.

Recent evidence challenges the conventional view of AR solely as a driver of proliferation, revealing its paradoxical capacity to induce cellular senescence and act as a tumor suppressor under specific conditions currently exploited in clinical trials [40, 41, 105, 106]. The circadian rhythm plays a crucial role in maintaining physiological homeostasis and is intimately involved in hormone production via the regulation of androgen biosynthesis. Disruptions in the circadian clock rhythm, whether due to lifestyle factors like shift work or molecular dysregulation, have been associated with increased cancer risk, including PCa [20–22, 133, 134]. We propose that a better understanding of the interplay of these factors may contribute to administering medical treatment with better outcomes for patients, reduced side effects, and less therapy resistance.

Altogether, the convergence of AR signaling, circadian rhythm, nutrition, and cellular senescence represents a novel and multifaceted regulatory axis in PCa biology. Despite advances in each pathway domain, our understanding of their integrated function remains limited. Uncovering how these pathways interact temporally and mechanistically could open new avenues for therapeutic interventions. Moving forward, a system-level approach that accounts for temporal regulation and crosstalk between these pathways will be essential for the development of more effective and personalized therapies for cancers including prostate.

## Data Availability

Not applicable.

## Abbreviations

AA: Atraric Acid
AR: Androgen receptor
BAT: Bipolar Androgen Therapy
Dar: Darolutamide
Enz: Enzalutamide
lncRNA: Long noncoding RNA
PCa: Prostate cancer
PSA: Prostate-specific antigen
SASP: Senescence associated secretory phenotypes
SAHF: Senescence associated heterochromatin foci
SADS: Senescence associated distension of satellites
SAL: Supraphysiological androgen levels
SA-β-Gal: Senescence associated beta galactosidase
TTFL: Transcription-translation feedback loop
